# Kidney organoid systems for studies of immune-mediated kidney diseases: challenges and opportunities

**DOI:** 10.1007/s00441-021-03499-4

**Published:** 2021-07-26

**Authors:** Melissa C. Stein, Fabian Braun, Christian F. Krebs, Madeleine J. Bunders

**Affiliations:** 1 Research Department Virus Immunology, Leibniz-Institute for Experimental Virology, Hamburg, Germany; 2 III. Department of Medicine, University Medical Center Hamburg-Eppendorf, Hamburg, Germany; 3 Division of Translational Immunology, III. Department of Medicine, University Medical Center Hamburg-Eppendorf, Hamburg, Germany; 4 Hamburg Center for Translational Immunology (HCTI), University Medical Center Hamburg-Eppendorf, Hamburg, Germany

**Keywords:** Kidney organoids, Tubuloids, Immune cells, Immune-mediated kidney diseases, Immune cell–epithelial cell interaction

## Abstract

Acute and chronic kidney diseases are major contributors to morbidity and mortality in the global population. Many nephropathies are considered to be immune-mediated with dysregulated immune responses playing an important role in the pathogenesis. At present, targeted approaches for many kidney diseases are still lacking, as the underlying mechanisms remain insufficiently understood. With the recent development of organoids—a three-dimensional, multicellular culture system, which recapitulates important aspects of human tissues—new opportunities to investigate interactions between renal cells and immune cells in the pathogenesis of kidney diseases arise. To date, kidney organoid systems, which reflect the structure and closer resemble critical aspects of the organ, have been established. Here, we highlight the recent advances in the development of kidney organoid models, including pluripotent stem cell-derived kidney organoids and primary epithelial cell-based tubuloids. The employment and further required advances of current organoid models are discussed to investigate the role of the immune system in renal tissue development, regeneration, and inflammation to identify targets for the development of novel therapeutic approaches of immune-mediated kidney diseases.

## Introduction

**Fig. 1:**
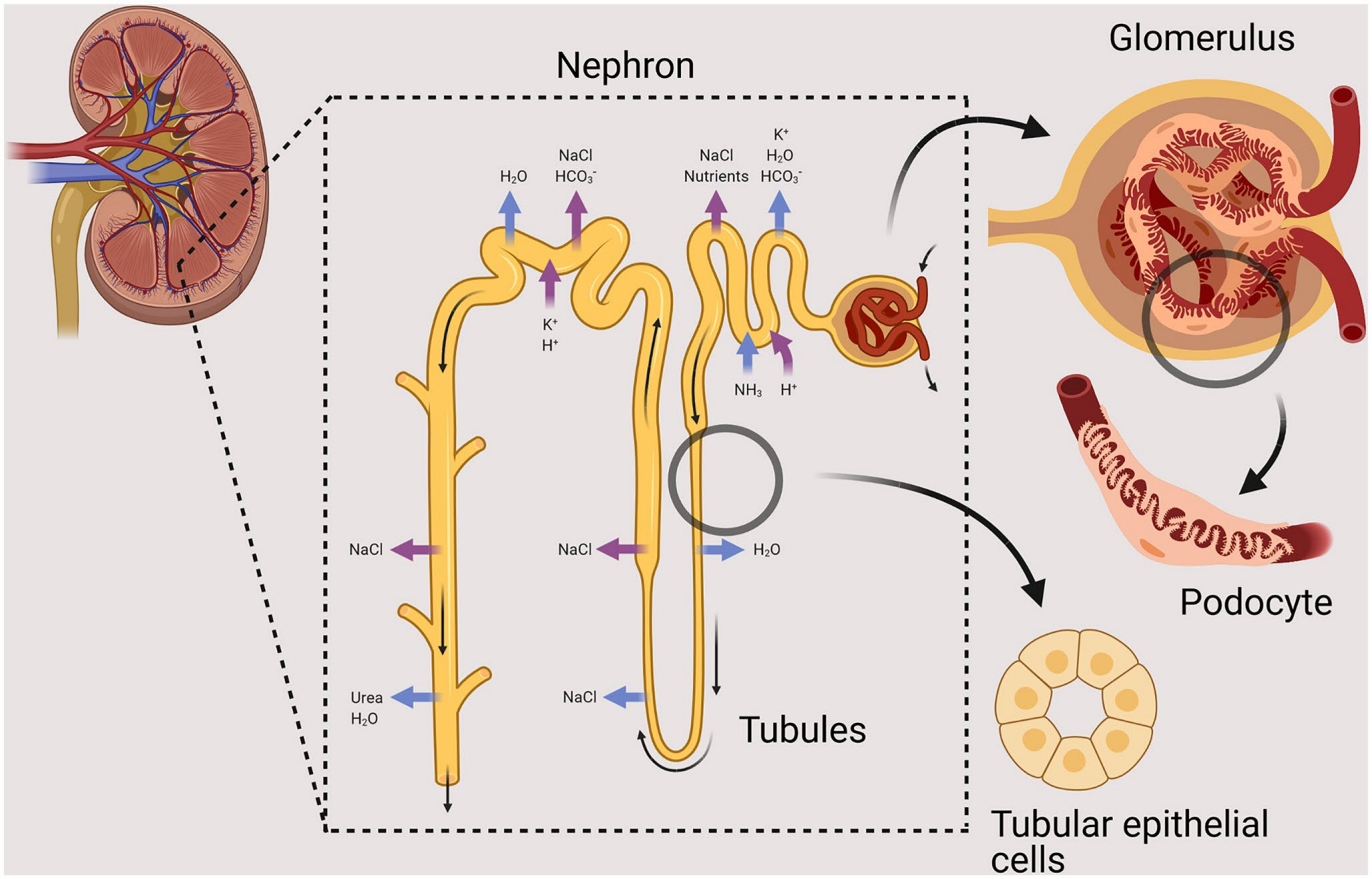
Kidney anatomy and physiology

Kidney diseases affect approximately 10% of the global population (Rewa and Bagshaw 2014; Ruiz-Ortega et al. 2020). In many of the diseases affecting the kidney, dysregulated immune responses are considered to importantly contribute to the pathogenesis. This is not surprising considering the kidney’s filtration function and related large epithelial surface, which facilitate interactions between immune cells and kidney epithelial cells (Fig. 1). Despite extensive research efforts and important discoveries in this field, the underlying mechanisms of several immune-mediated kidney diseases are not yet fully understood. Until recently, studies investigating kidney diseases had to rely mostly on transformed cell lines or animal models, which not always accurately reflect the human in vivo situation with regard to gene expression or genetic background. With recent progress in the development of organoids, a new technology to model human tissues in vitro has become available (Clevers 2016; Bar-Ephraim et al. 2020). As intestinal organoids were one of the first models developed (Sato et al. 2009), the earliest technological advances to study immune cell-epithelial cell crosstalk were explored using intestinal organoids (Bar-Ephraim et al. 2020). These pioneering studies identified, for example, mechanisms of tumor-reactive T cell effector functions based on organoid-lymphocyte co-cultures (Cattaneo et al. 2020) as well as T cell-mediated regulation of human intestinal development and inflammation (Schreurs et al. 2019). To date, there are several sophisticated kidney organoid systems, which recapitulate human kidney tissue in vitro and provide novel opportunities to study immune cell-epithelial cell crosstalk in the kidney (Freedman et al. 2015; Morizane et al. 2015; Takasato et al. 2015; Taguchi and Nishinakamura 2017; Przepiorski et al. 2018; Schutgens et al. 2019; Kumar et al. 2019). The generation of these multicellular organoid models was supported by the increasing understanding of nephrogenesis and renal tissue regeneration. New developments and adaptions of many organoid models continue to be reported (van den Berg et al. 2018; Homan et al. 2019; Kumar et al. 2019; Gijzen et al. 2021). In this perspective, we discuss the potential avenues to use induced pluripotent stem cell (iPSC)-derived kidney organoids and tubuloids in studying immune-mediated kidney diseases and provide a better understanding of the immune system’s role in renal tissue development, regeneration, and inflammation. We further discuss the challenges facing the use of organoids to accurately model immune-mediated kidney diseases and technical advances of this evolving technology that are still required for broad implementation in this context.

## Organoids: the development of in vitro tissue models

Within the last 10 years organoids, three-dimensional structures generated from stem cells, which self-organize and differentiate into organ specific cell types, have emerged as an innovative in vitro model to study fundamental principles of human biology (Yousef Yengej et al. 2020). Organoids furthermore offer unparalleled opportunities to investigate a wide range of pathologies and model disease of individual patients. Organoids can be used as diagnostic tools or to test individual therapeutic options to tailor the treatment to a patient’s genotype and phenotype (Clevers 2016; Dijkstra et al. 2018; Hollywood et al. 2020). Eiraku and Sasai were the first to describe a culture method resulting in the formation of multicellular, differentiated, three-dimensional cortical tissue, which arose from embryonic stem cells (Eiraku et al. 2008). In 2009, Sato and colleagues developed an organoid system using isolated primary epithelial cells derived from murine intestines and established culture conditions, which emulate the physiological environment during intestinal tissue regeneration. Their approach facilitated the in vitro propagation of Leucine-rich repeat-containing G-protein coupled receptor 5-positive (LGR5^+^) adult stem cells (ASCs) and paved the way for the generation of ASC-derived organoids (Sato et al. 2009). At present, organoids can either be derived from embryonic stem cells, iPSCs, or organ specific ASCs.

The development of iPSCs has been an important technical breakthrough, which provided an alternative to the use of blastocysts-derived embryonic stem cells (Takahashi et al. 2007). iPSCs are generated by reprogramming primary somatic cells, for example, fibroblasts, peripheral blood mononuclear cells (PBMCs), or urinary cells into embryonic stem cell-like cells (Takahashi et al. 2007; Sommer et al. 2012). After transformation, iPSCs can be subsequently differentiated towards precursor cells from all three germ layers (Takahashi et al. 2007; Zhou et al. 2011; Sommer et al. 2012). In the last decade, this technique has been used to develop organoid models for many tissues including, but not limited to, the brain (Lancaster et al. 2013), liver (Takebe et al. 2013), intestine (Spence et al. 2011), and kidney (Morizane et al. 2015; Takasato et al. 2015; Taguchi and Nishinakamura 2017; Howden and Little 2020).

ASC-derived organoids on the other hand are generated from isolated, tissue-specific cells. ASCs are cultured without reprogramming, and by the addition of carefully balanced Wnt-activators and tissue-specific growth factors, the cells are coaxed to form organoids (Clevers 2016). At present, ASC-derived organoid models exist for the intestine (Sato et al. 2009), liver (Huch et al. 2015), lung (Sachs et al. 2019), and multiple other organs (Huch et al. 2013; Chua et al. 2014; Kessler et al. 2015; Lugli et al. 2016). Recently, a similar approach for the generation of primary kidney tubular epithelial organoids called tubuloids was developed (Schutgens et al. 2019).

In conclusion, organoids recapitulate structural and functional characteristics of an organ, which makes them robust in vitro models of human tissue. This has led to their rapid implementation in research. Today, organoids are employed in translational approaches for studying infectious diseases (Schutgens et al. 2019; Sachs et al. 2019; Monteil et al. 2020; Wysocki et al. 2021), cancer (Weeber et al. 2015; Calandrini et al. 2020; Cattaneo et al. 2020), and hereditary diseases (Freedman et al. 2015; Schutgens et al. 2019; Sachs et al. 2019). They furthermore represent a promising technology for personalized and regenerative medicine (Clevers 2016; Dijkstra et al. 2018; Hollywood et al. 2020).

## iPSC-derived kidney organoids

### The development of iPSC-derived kidney organoids

The development of organoids recapitulating the kidney’s individual multicellular architecture builds upon our understanding of nephrogenesis and formation of the three respective precursors pronephros, mesonephros, and metanephros during gestation (Little et al. 2010). The complexity of the kidney with the nephron comprising of over 20 distinct cell types and the intricate reciprocal interactions of the metanephric mesenchyme and ureteric bud during nephrogenesis (Little and Combes 2019; Yousef Yengej et al. 2020) have delayed the development of kidney organoids. In 2014, Taguchi and colleagues achieved the successful differentiation of human iPSCs into metanephric mesenchyme. Using phasic Wnt-stimulation and the timed addition of growth-factors mimicking physiological nephrogenesis, they induced metanephric progenitors to give rise to kidney tubules and glomeruli (Taguchi et al. 2014). In 2015, Takasato and colleagues were able to distinguish the distinct temporospatial origins of the collecting duct and kidney mesenchyme progenitors, and optimized kidney organoid formation by actively triggering nephrogenesis through reciprocal interactions of ureteric epithelial cells and metanephric mesenchymal cells. This resulted in the formation of intricate, multicellular kidney organoids containing nephron-like structures with an associated collecting duct network surrounded by renal interstitium and endothelial cells (Takasato et al. 2015). At the same time, other groups developed alternative approaches to generate iPSC-derived kidney organoids based on the induction of nephron progenitor cells from late-stage mid-primitive streak. Subsequently, these progenitor cells spontaneously formed nephron-like structures reflecting all key epithelial derivates of metanephric mesenchyme (Morizane et al. 2015). Further progress was achieved by selective induction of nephron progenitors, ureteric bud, and stromal progenitors followed by the reassembly of all three lineages to develop more complex kidney organoids (Taguchi and Nishinakamura 2017). These approaches are however costly and time-consuming; therefore, more recently efforts have been made to increase the scalability and reduce costs for the generation of iPSC-derived kidney organoids (Przepiorski et al. 2018; Kumar et al. 2019). Here, the culture conditions were modified to allow formation of kidney organoids in suspension culture, which increased the yield of cellular structures and decreased the overall costs (Kumar et al. 2019).

### iPSC-derived kidney organoids: characterization and assessment

iPSC-derived kidney organoids have been characterized to assess their value for disease modeling, drug- and toxicity-screening, and a new tool to further advance personalized medicine. Especially the fidelity of iPSC-derived kidney organoids to robustly replicate normal kidney development with particular attention paid to cellular identity, and development has been elaborately investigated. Applying single-cell RNA-sequencing approaches revealed a high level of congruence between kidney organoids and the human fetal kidney regarding gene expression within endothelial, stromal, and nephron cell types (Combes et al. 2019). Further, distinct nephron segments were identified by protein expression and included cells with profiles of podocytes, early proximal tubule cells, early distal tubule cells, and collecting duct epithelial cells (Takasato et al. 2015). Compared to conditionally immortalized human podocyte cell lines, podocytes in kidney organoids show appropriate basolateral polarity and gene expression profile better reflecting the in vivo situation (Takasato et al. 2015; Hale et al. 2018; Tanigawa et al. 2018). To assess the capability of iPSC-derived kidney organoids for disease modeling and drug-screening, the maturation of the proximal tubule has been analyzed. There was evidence for cubilin-mediated endocytosis in proximal tubule cells, which supports an advanced functional cell maturation. In addition, the potential for nephrotoxicity screening was tested with cisplatin, which induced dose-dependent apoptosis in proximal tubular cells mirroring the effect in vivo (Takasato et al. 2015). Thus, iPSC-derived kidney organoids represent a complex model of the structural and in part functional key elements of the kidney.

### iPSC-derived kidney organoids: challenges and adaptions

Despite these promising advances challenges remain, which hamper the general use of iPSC-derived kidney organoids in clinical research. One of the most critical limitations of kidney organoids is the fact that they recapitulate the developing human fetal kidney (Takasato et al. 2015). Even though nephron formation is completed before birth (Little et al. 2010), comparisons with a fully functional postnatal kidney are limited. Evidence for the presence of cell-type specific ligands and receptors mediating human nephrogenesis (Combes et al. 2019) as well as markers of terminal differentiation are still missing (Wu et al. 2018). An additional limitation of iPSC-derived kidney organoids is the absence of certain cell types, which are present in vivo in the human kidney. Although there is evidence for vasculature formation and development of endothelial networks in iPSC-derived kidney organoids (Morizane et al. 2015; Takasato et al. 2015), these models do not form a fully matured glomerulus. This cannot be solved by longer periods of differentiation, as such protocols were associated with a decline of endothelial cells as well as an overall decrease of nephron cells (van den Berg et al. 2018; Przepiorski et al. 2018). It has been proposed that the lacking blood supply combined with the absence of immune cells and organized neurons contributes to the limited functional maturation, growth, and longevity of kidney organoids (Little and Combes 2019).

To address the limited maturation, efforts have been made to establish a functioning blood supply to kidney organoids. iPSC-derived kidney organoids were transplanted under the renal capsule of immune-compromised mice. After transplantation, the organoids grew significantly in size while maintaining glomerular and tubular structures. With time, the transplanted glomerular structures were perfused as the ingrowing renal vasculature of the host sprouted capillaries invading the premature glomeruli. The transplanted organoids formed a glomerular basement membrane and the tubule epithelium formed a single monolayer with a well-developed apical brush border and luminal microvilli and cilia (van den Berg et al. 2018). In another approach to expand the organoid’s endogenous endothelial cellular networks, iPSC-derived kidney organoids were cultured on 3D printed perfusable chips to generate a fluidic shear stress. Interestingly, kidney organoids exposed to high fluidic shear stress developed a more mature vasculature than organoids cultured under static or low fluidic shear stress conditions. Altogether, the exposure to controlled flow of fluids facilitated the formation of vasculature and enhanced the maturity of tubular epithelium (Homan et al. 2019). These studies reveal that establishing a functional vascularization of kidney organoids or application of fluid flow can promote progressive morphogenesis and maturation and will increase the utility of kidney organoids as precise in vitro models for the study of kidney diseases in the future. However, it should be mentioned that the transfection and transformation of cells to induce pluripotency has additional challenges. iPSCs are vulnerable to tumorigenicity, whereas the removal of epigenetic marks by reprogramming impedes the use of iPSC-derived kidney organoids for non-heritable kidney diseases and related personalized medicine (Lee et al. 2013). Therefore, efforts have been made to generate kidney organoids from patient-derived cells similar to previous protocols for the generation of ASC-derived organoids of the liver (Huch et al. 2015) or intestine (Sato et al. 2009); these primary epithelial cell-based tubuloids are described below.

## Tubuloids

### Tubuloids: establishment, characterization, and assessment

Recently, Schutgens and colleagues achieved the generation of patient-derived primary renal tubular epithelial organoids and termed them tubuloids as they represent distinct tubule segments of the nephron but lack glomerular cells. The renal epithelial cells required to generate tubuloids are obtained from kidney tissue or urine (Schutgens et al. 2019). Especially use of exfoliated renal epithelial cells from urine is a promising non-invasive technique for organoid generation. The protocol harnesses the kidney’s mechanisms for tissue repair and regeneration by promoting the dedifferentiation of tubular epithelial cells into a progenitor state as well as proliferation of progenitor cells into segment specific tubule epithelium (Berger et al. 2014; Kang et al. 2016). Isolated cells are seeded in a solubilized basement membrane preparation rich in extracellular matrix proteins and cultured in a medium supplemented with Rspo1-conditioned medium and growth-factors (FGF10, EGF). These components have been described to promote renal tissue repair in vivo (Poladia et al. 2006; Watanabe et al. 2007; Lancaster et al. 2009; Xu et al. 2009)*.* Consequently, tubuloid culture conditions give rise to epithelial tubule cells recapitulating tissue regeneration and repair.

Tubuloids have been developed more recently than iPSC-derived kidney organoids. Therefore, extensive characterization of these models is still lacking. Yet, based on single-cell RNA-sequencing analyses, gene expression profiles of distinct nephron segments were detected including the proximal tubule, loop of Henle, distal tubule, and collecting duct. Further, tubuloids contained cells with multilineage potential as tubuloid lines established from a single-cell expressed marker genes of different nephron segments (Schutgens et al. 2019). Gene expression of hallmarks of endothelial and interstitial cells were furthermore lacking in tubuloids. Tubuloids displayed appropriate function of the proximal tubule xenobiotics efflux pump ABCB1 (P-glycoprotein), indicating functional maturation of the generated tubule cells (Schutgens et al. 2019). Further studies are required to precisely characterize the cells in tubuloids and their reflection of the in vivo human tubule.

### Tubuloids: challenges and adaptions

Although tubuloids have the advantage to be derived from ASCs, the absence of stromal populations, podocytes, and vascularization limits their use for modelling diseases in which these renal structures play an important role, e.g., glomerulonephritis. Further, tubuloids develop as cystic structures and do not display tube-like nephron formation. At present, tubuloid culture protocols are optimized to generate proximal tubule cells (Schutgens et al. 2019). Adaptations of the protocols are required to allow the formation of tubuloids representing the in vivo distribution of all distinct nephron segments with their associated transporter proteins and enzymes. Recently, tubuloids have been cultured on microfluidic chips to mimic the renal microenvironment and promote tubule formation (Schutgens et al. 2019; Gijzen et al. 2021). Under these conditions, tubuloids formed leak-tight, perfusable, differentiated kidney tubules. This approach facilitates the engineering of more complex tissue structures. Further, the exposure to fluid flow enables continuous media refreshment of tubuloid cultures (Schutgens et al. 2019; Gijzen et al. 2021). Building on these recent developments will allow the generation of more complex tubuloid models.

## iPSC-derived kidney organoids and tubuloids: unique tissue models

**Fig. 2:**
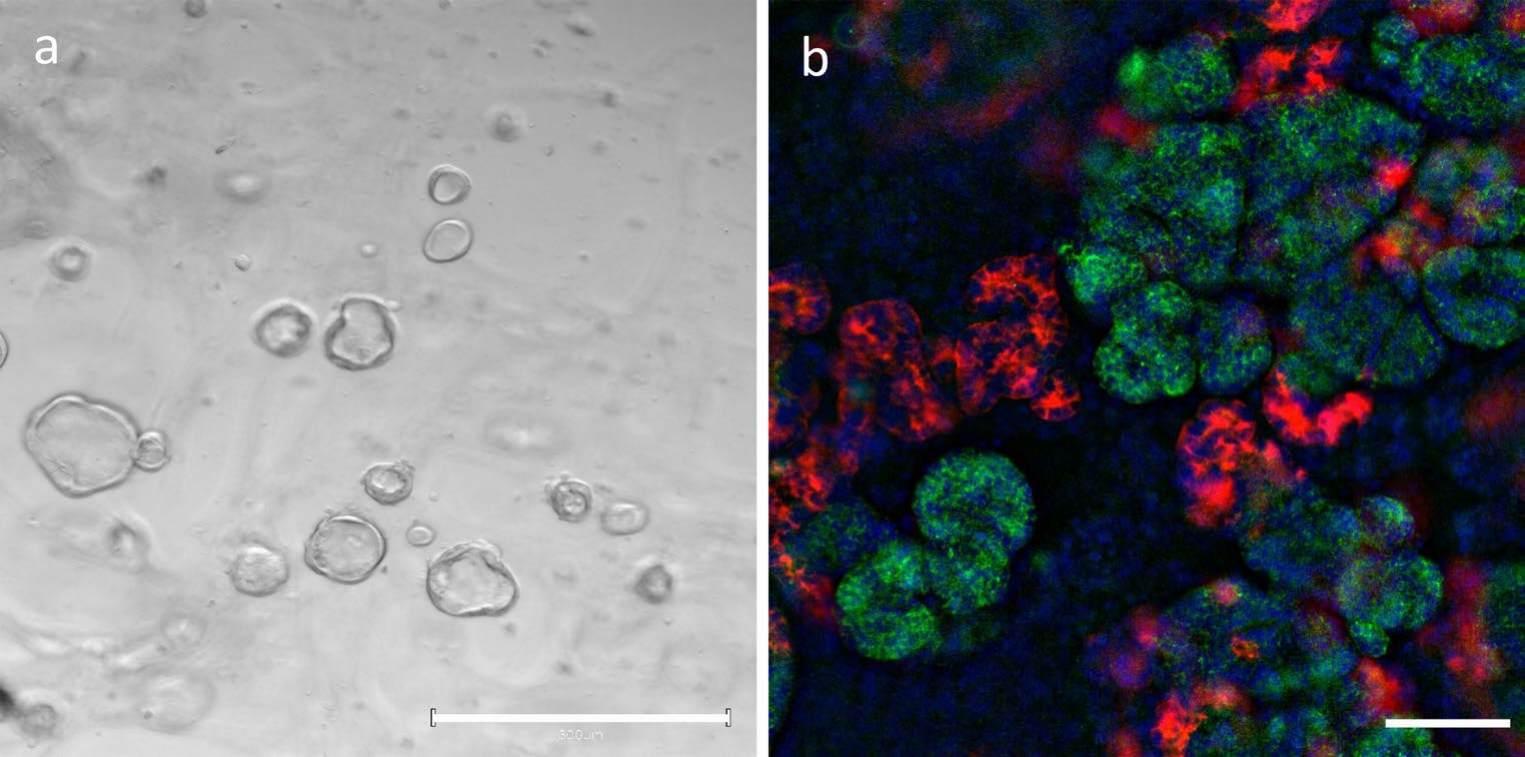
Tubuloids and iPSC-derived kidney organoids: **a** Urine-derived tubuloids with typical cystic morphology; scale bar: 300 µm. **b** Immunofluorescence staining of iPSC-derived kidney organoids red: LTL staining = proximal tubular cells, green: Nephrin, blue: Dapi = nuclei; scale bar: 20 µm

For the successful employment of organoids to investigate immune-mediated kidney diseases, the advantages and drawbacks of iPSC-derived kidney organoids and tubuloids need to be assessed to design meaningful studies (Table 1). The main differences between the iPSC-derived organoids and tubuloids are that iPSC-derived kidney organoids recapitulate nephrogenesis, whereas tubuloids model renal tissue regeneration and repair of the renal tubule. iPSC-derived kidney organoids self-organize into nephron-like structures and exhibit greater cellular complexity (Morizane et al. 2015; Takasato et al. 2015; Taguchi and Nishinakamura 2017). Tubuloids do not form a glomerulus, lack podocytes, endothelial, and interstitial cells, which can all be found in iPSC-derived kidney organoids (Schutgens et al. 2019) (Fig. 2). But, as mentioned above, the reprogramming of differentiated cells into iPSCs is often associated with genomic instability and the models have a limited life-span (Lee et al. 2013; van den Berg et al. 2018; Przepiorski et al. 2018). Further, iPSC-derived kidney organoids suffer from fibrotic changes over time, resulting in the proliferation of MEIS1/2/3^+^ interstitial cells and loss of proximal tubule function (Przepiorski et al. 2018). Tubuloids on the other hand can be cultured for more than 6 months and passaged over 20 times, while maintaining genomic stability. This highlights the genetic robustness of tubuloids and allows long-term expansion (Schutgens et al. 2019). Altogether, both organoid types have their unique advantages and challenges that can be harnessed and need to be taken into consideration to design studies to model immune-mediated kidney diseases.

**Table 1:** Characteristics of iPSC-derived kidney organoids and tubuloids

	iPSC-derived kidney organoids	Tubuloids
Source	Induced pluripotent stem cells	Patient-derived cells (tissue or urine)
Cellular components	Podocytes, tubular epithelial cells, endothelial and stromal progenitors	Tubular epithelial cells
Structure	Organized, nephron-like structure	Cystic or dense structure
Culture period	Maintenance for 3 weeks, followed by fibrotic changes and genomic instability	Stable expansion and culture over 6 months

## Use of organoid models beyond stem cell biology

In recent publications, kidney organoids and tubuloids were primarily used to assess developmental and genetic diseases (Freedman et al. 2015; Forbes et al. 2018; Hale et al. 2018; Tanigawa et al. 2018; Schutgens et al. 2019). Although immune cells are detected in basically all human tissues including the kidney, basic in vitro organoid models lack immune cells. To advance organoid models, protocols for the co-culture of organoids and immune cells have been developed. These protocols provide a new platform to investigate the intricate epithelial cell–immune cell crosstalk. However, due to the relative novelty of kidney organoids and tubuloids, co-cultures with immune cells have not been established yet. Nevertheless, the latest advances in other fields, which are summarized below, using organoid-immune cell co-cultures can aid the development of new protocols (Rogoz et al. 2015; Biton et al. 2018; Dijkstra et al. 2018; Sachs et al. 2019; Schreurs et al. 2019). Recently, it has been shown that tumor-reactive CD8^+^ T cells can be expanded when co-cultured with tumor organoids and assessed for their anti-tumor responses, providing proof-of-concept for developing patient-specific T cell-based therapies (Dijkstra et al. 2018). Similarly, tumor organoid–T cell co-cultures could be used to assess the efficacy of individual drugs in the presence of immune cells to tailor treatment to the individual patient for example in children suffering from kidney cancer. A pediatric kidney tumoroid bank has been established amongst others for this purpose (Calandrini et al. 2020). Further, the severe acute respiratory syndrome corona virus type 2 (SARS-CoV-2) pandemic has underlined the value of kidney organoids in the field of infectious diseases research, as kidney organoids expressing ACE2 (angiotensin converting enzyme 2) were used as in vitro models for SARS-CoV-2 infection. The addition of human recombinant soluble ACE2 reduced viral infection of kidney organoids and blocked early stages of infection, demonstrating the importance of ACE2 for infection of SARS-CoV-2 (Monteil et al. 2020; Wysocki et al. 2021). Tubuloids have furthermore been infected with BK virus, a member of the polyoma-virus family, which resulted in intranuclear basophilic viral inclusions as seen in BK nephropathy (Schutgens et al. 2019). Together these studies illustrate the use of kidney organoid models for infectious disease research. To develop models to study immune-mediated kidney diseases, a deep understanding of immune cells and inflammatory pathways in parenchymal cells of the kidney is needed. Here, we summarize factors contributing to the intricate relationship between immune cells and epithelial cells of the kidney. This is followed by a prospect of potential applications of kidney organoids to investigate the role of the immune system in kidney diseases, renal tissue development and regeneration. Lastly, we discuss improvements required for the establishment of iPSC-derived kidney organoids and tubuloids to proficiently recapitulate the role of immune responses in the pathogenesis of kidney diseases and application for regenerative medicine.

## Renal immune cell populations in health and disease

Due to the kidney’s high blood perfusion, a large number of immune cells, antigens, and signaling molecules of immune cells such as cytokines and immunoglobulins circulate renal tissue with the potential to interact with renal epithelial cells (Murray and Paolini 2020). The excretion of harmful metabolic waste products and the clearing of cytokines as well as bacterial products including toxins by the kidney emphasize the interplay between kidney function and immunological homeostasis (Tecklenborg et al. 2018). Under physiological circumstances, the kidney contains large numbers of immune cells (Soos et al. 2006), which—in collaboration with renal epithelial cells—protect the body from pathogens (Krebs et al. 2020). However, when this delicately balanced system is dysregulated leukocytes can be further attracted and activated, causing tissue damage in immune-mediated kidney diseases (Banas et al. 2008; Beck et al. 2009; Krebs et al. 2016).

Under physiological conditions, the intrarenal immune system largely consists of macrophages and dendritic cells (DCs) (Hume and Gordon 1983; Kaissling and Le Hir 1994; Soos et al. 2006). Renal macrophages (CD68^+^CD11b^+^MHCII^+^) reside in the renal interstitium as well as in glomerular and tubulointerstitial vessels (Kaissling and Le Hir 1994; Ferenbach and Hughes 2008; Segerer et al. 2008). Renal DCs (CD11b^+^CD11c^+^BDCA-1^+^DC-SIGN^+^) are restricted to the tubulointerstitial space, where they encounter a large amount of circulating antigens and function as sentinels (Dong et al. 2005; Soos et al. 2006; Ferenbach and Hughes 2008; Segerer et al. 2008; Nelson et al. 2012). The majority of the filtered, soluble antigens are of endogenous origin, such as hormone peptides or innocuous food-derived peptides. Renal DCs usually promote peripheral tolerance to these antigens to maintain overall immune homeostasis (Lukacs-Kornek et al. 2008; Gottschalk et al. 2013; Tecklenborg et al. 2018). Specialized renal DC populations express PD-L1 (program-death 1 ligand), which upon antigen presentation in renal lymph nodes to PD1^+^ CD8^+^ CTLs (cytotoxic T cells) induces apoptosis of the CTL (Gottschalk et al. 2013). DCs resident in renal lymph nodes furthermore induce cross-tolerance against filtered antigens, which results in the induction of a CD44^+^CD25^−^CD62L tolerized CD8^+^ T cell phenotype (Lukacs-Kornek et al. 2008). Together, the presentation of antigens by renal DCs to CD8^+^ T cells normally promotes peripheral tolerance against circulating peptides.

Notably, the expression of inhibitory molecules such as PD-L1 is not restricted to renal macrophages and DCs. Recent studies demonstrate that proximal tubular epithelial cells express molecules involved in immune tolerance mediation such as PD-L1, HLA-G, and IDO (Indoleamine 2, 3-dioxygenase) (Wilkinson et al. 2011; Kassianos et al. 2013; Sampangi et al. 2015). The pathways regulated by these molecules are of great interest, as they contribute to immune homeostasis; however, upon dysregulation, they can contribute to inflammation (Hou et al. 2009; Wilkinson et al. 2011; Gottschalk et al. 2013; Naji et al. 2014). iPSC-derived kidney organoids and tubuloids containing these cell types can be used to assess the role of parenchymal cells in mediating tolerance in the kidney and how these mechanisms may be perturbed in kidney diseases.

## Modeling immune-mediated kidney diseases with kidney organoids and tubuloids

The critical role of the immune system in kidney homeostasis is exemplified by the array of kidney diseases where dysregulated immune responses importantly contribute if not drive the pathogenesis (Kurts et al. 2013; Krebs and Panzer 2018). Immune-mediated kidney diseases can be categorized according to the localization of renal injury, involvement of immune cell subtypes and clinical course. Systemic diseases include anti-neutrophil cytoplasmic antibodies (ANCA)-associated vasculitis and systemic lupus erythematosus (SLE) that display renal inflammation in the glomerulus and in the tubulointerstitial compartment but also show extra-renal inflammation (Bohle A 1994; Kurts et al. 2013). In primary glomerulonephritis such as IgA nephropathy, membranous nephropathy, anti-glomerular basement membrane (GBM) nephritis, and primary focal segmental glomerulosclerosis (FSGS), the disease is limited to the kidney (Kopp et al. 2020; Braun et al. 2021). While in these diseases, the immune system seems to be the main driver of pathology, it is important to note that leukocytes interact with various parenchymal cells in the kidney. To this end, the tubule system plays a critical role in mediating signals that result in tissue damage through both direct and indirect inflammatory effects. Here, we discuss these diseases, describe the recognized contribution of dysregulated immune responses, and how organoids can aid to further understand the complex underlying mechanisms leading to progressive glomerular and tubulointerstitial destruction.

### Rapid progressive glomerulonephritis

The most aggressive form of glomerular inflammation is seen in rapid progressive glomerulonephritis. The underlying diseases that result in this clinical phenotype have been classified into three categories: type 1 (immune-depositions along the GBM): anti-GBM disease and Goodpasture’s syndrome with renal and pulmonary involvement; type 2 (immune-complex glomerulonephritis): numerous infectious and inflammatory diseases including lupus nephritis; and type 3 (pauci-immune glomerulonephritis): ANCA-associated glomerulonephritis (granulomatosis with polyangiitis, microscopic polyangiitis, eosinophilic granulomatosis with polyangiitis) (Couser 1988).

Besides circulating immune complexes and autoantibodies, dysregulated T cell responses mediate severe inflammation and tissue damage in autoimmune kidney diseases (Riedel et al. 2021). CD4^+^ T cells have been shown to drive rapid progressive glomerulonephritis as seen in ANCA-associated glomerulonephritis, anti-GBM nephritis, and lupus nephritis. Especially the role of IL-17 producing CD4^+^ T_H_17 cells has gained increasing attention in kidney inflammation (Krebs et al. 2017; Krebs and Panzer 2018; Krohn et al. 2018; Riedel et al. 2021). An increased T_H_17 response is associated with a more severe course of glomerulonephritis and tubulointerstitial injury (Krebs et al. 2017). IL-17C deficiency as well as deficiency of the respective IL-17 receptor E furthermore ameliorates tissue injury and kidney function in mouse models of crescentic glomerulonephritis and lupus nephritis (Krohn et al. 2018). Tubular injury and inflammation was attenuated upon IL-17C neutralization as well as in a kidney injury IL-17 receptor E knockout-mouse model (Wang et al. 2020). Tubulointerstitial macrophage and T cell infiltration are significantly reduced in nephritic Il-17c^−^/^−^ mice (Krohn et al. 2018). IL-17A and F production by infiltrating T_H_17 cells has been shown to activate tubular epithelial cells to produce chemokines attracting neutrophils as well as CXCL9 expression that mediates the recruitment of Th1 cells and drives the later stages of renal injury in experimental glomerulonephritis (Paust et al. 2012; Disteldorf et al. 2015; Krebs and Panzer 2018). iPSC-derived kidney organoids and tubuloids can be used to assess T cell recruitment in an organoid model. For these studies urine-derived tubuloids from patients could be employed to investigate disease-specific immune cell migration in the renal microenvironment and assess potential interventional therapies. Both iPSC-derived kidney organoids and tubuloids furthermore provide a human cell-based system to determine T helper cell polarization by renal epithelial cells in kidney diseases even at the individual patient level. Recently, there have been studies reporting the successful co-culture of intestinal organoids with T cells and the subsequent analysis of epithelial cell-mediated T cell differentiation (Rogoz et al. 2015; Biton et al. 2018). Activated peripheral T cells changed their features towards a phenotype resembling intraepithelial lymphocytes upon co-culture with intestinal organoids (Rogoz et al. 2015). In a similar approach, iPSC-derived kidney organoids and tubuloids could be employed to assess the direct interactions between renal epithelial cells and T_H_17 cells and their role in driving dysregulated T helper responses in kidney diseases.

### Lupus nephritis

Approximately 50% of SLE patients develop lupus nephritis, which is associated with the dysregulation of several TLRs (toll-like receptors) including TLR4, TLR7, and TLR9 (Christensen et al. 2006; Liu et al. 2006; Lee et al. 2008; Smith 2009; Almaani et al. 2017). Podocytes and renal tubular epithelial cells constitutively express TLRs and are able to recognize pathogen-associated molecular patterns (PAMPs) of bacteria or viruses (Wolfs et al. 2002; Tsuboi et al. 2002; Banas et al. 2008). TNF-α and IFN-γ can further increase the expression of TLR2 and TLR4 by renal epithelial cells, amplifying the cellular responses to PAMPs (Wolfs et al. 2002). Activation of TLRs in podocytes and renal tubular epithelial cells induces the production of proinflammatory chemokines and cytokines, which attract and activate immune cells including T and B cells (Tsuboi et al. 2002; Banas et al. 2008; Demmers et al. 2013). Functional TLR expression is maintained by epithelial cells in other organoid systems such as the gut (Price et al. 2018), and although this needs to be confirmed, it likely also applies to cells in tubuloids and iPSC-derived kidney organoids. Both kidney organoid systems could therefore represent robust human cell-based models to study the activation of innate responses in renal epithelial cells and the consequences for immune cell migration and their activation in the renal microenvironment.

Dysregulated T and B cell responses importantly contribute to the pathogenesis of lupus nephritis (Munroe and James 2015). Several MHC haplotypes (HLA-DR2, HLA-DR3,and HLA-DR15) have been identified to increase the risk of SLE and lupus nephritis (Graham et al. 2007; Chung et al. 2014; Niu et al. 2015). Until now, the role of these HLA molecules has been linked to comprised immune tolerance to nuclear autoantigens (Yellin et al. 1997; Clynes et al. 1998; Goodnow 2007; Xu et al. 2017). However, studies suggest that epithelial cells such as podocytes and renal tubular cells also express HLA molecules and even regulate T cell activation (Wuthrich et al. 1989; Frasca et al. 1998; Banu and Meyers 1999; Yuan et al. 2020). Podocytes express increased numbers of MHC I and II molecules upon stimulation with cytokines (Henny et al. 1986; Goldwich et al. 2013; Li et al. 2020). In vitro and in vivo studies have shown that CD8^+^ T cells as well as CD4^+^ T cells can be activated by CD56^+^ CD80^+^ podocytes presenting antigens on MHC I or MHC II molecules (Goldwich et al. 2013; Li et al. 2020) and may contribute to the induction of dysregulated responses. In line with these observations in podocytes, HLA class I and class II molecules are increased on tubular epithelial cells in the context of kidney transplant rejection (Henny et al. 1986; Bishop et al. 1988). Therefore, podocytes and tubular epithelial cells may play an important role in the activation of the adaptive arm of the immune system, in particular upon kidney injury (Banu and Meyers 1999; Starke et al. 2010; Wilkinson et al. 2011; Goldwich et al. 2013). Organoids have been used in other contexts to study MHC-T cell receptor-mediated interactions between epithelial cells and T cells, and it was shown that intestinal stem cells expressing MHC II are non-conventional antigen-presenting cells (Biton et al. 2018). These findings bear similarities to the expression of MHC I and MHC II on renal epithelial cells (Goldwich et al. 2013; Li et al. 2020). It remains to be determined whether MHC I and II expression by podocytes in iPSC-derived kidney organoids reflects their expression by podocytes in vivo; however, the potential to study T cell–podocyte interactions in an autologous system using blood-derived PBMCs from patients would importantly contribute to our understanding of these interactions in kidney diseases, including SLE.

### IgA nephropathy

IgA nephropathy is the most common form of primary glomerulonephritis (Stanley and Deng 2020), characterized by circulating immune complexes that are trapped in the filtration barrier (Kurts et al. 2013; Tecklenborg et al. 2018). Critical in the pathogenesis of IgA nephropathy is galactose-deficient IgA1, which elicits an autoantibody response against N-acetylgalactosamine epitopes on galactose-deficient IgA1 and formation of immune complexes (Berthoux et al. 2012). The deposition of immune complexes further activates the complement system, and induces cytokine and chemokine production by mesangial cells (Liu et al. 2017). Studies revealed that the transferrin receptor (CD71) can function as mesangial cell IgA receptor and facilitates immune complex deposition (Haddad et al. 2003; Moura et al. 2005; Tamouza et al. 2007). More recently, glomerular ß-1,4-galactosyltransferase 1 has been identified as a mesangial receptor that binds the Fc portion of IgA and blocking ß-1,4-galactosyltransferase 1 resulted in a significantly reduced IL-6 secretion (Molyneux et al. 2017). Early mesangial cells expressing platelet-derived growth factor receptor A (PDGFRA^+^) have been detected in iPSC-derived kidney organoids (Takasato et al. 2015) and provide the opportunity to assess the role of mesangial cells in IgA nephropathy. However, as current studies have not focussed mesangial cells, their induction and characterization in iPSC-derived kidney organoids lack behind our knowledge of podocytes or tubule cells. Further studies are needed to assess whether iPSC-derived models provide a viable system to model IgA nephropathy.

### Glomerular antibody deposition

Antibody deposition at the glomerular basement membrane can result in membranous nephropathy that is characterized by high proteinuria and a granular pattern of glomerular immune deposits. Autoantibodies against antigens expressed by healthy podocytes including the PLA2R (M-type phospholipase A2 receptor) (Beck et al. 2009) or Thrombospondin type-1 domain containing 7 (Tomas et al. 2014) are detected in the majority of patients with membranous nephropathy (Tomas et al. 2021). However, in some cases, the autoantigen is still elusive. In anti-GBM nephritis, autoantibodies bind to mainly two epitopes on the α3 chain of the non-collagenous-1 (NC1) domain of type IV collagen and form linear IgG deposits along the GBM causing rapidly progressive glomerulonephritis (Pedchenko et al. 2010; Olaru et al. 2013). Studies assessing the expression of these antigens in iPSC-derived kidney organoids could provide a new platform to dissect the antibody-mediated and immune cell-mediated mechanisms contributing to the pathogenesis of idiopathic membranous nephropathy and anti-GBM nephritis. Otherwise, transgenic expression of the aforementioned antigens could prime podocytes in iPSC-derived kidney organoids for the subsequent treatment and assessment of the effects of sera derived from membranous nephropathy and anti-GBM patients exert (Petrosyan et al. 2019). Similar experiments could be envisioned to determine the effects of the proposed circulation permeability factor(s) resulting in the development of primary FSGS (Braun et al. 2021).

### Interstitial nephritis and modelling kidney injury with organoids and tubuloids

In addition to the activation of inflammatory pathways by PAMPs, tubular epithelial cells are able to secret a range of proinflammatory cytokines upon ischemia further activating neighbouring immune cells. Renal DCs in return can produce chemokines attracting additional immune cells, which has been shown to contribute to neutrophil-dependent acute kidney injury (Leemans et al. 2005; Wu et al. 2007; Allam et al. 2012). Further, tubular epithelial cell injury mediated by non-infectious crystal formation can induce sterile inflammation (Thongboonkerd 2019). Tubuloids have recently been plated in a “organ-on-a-chip” platform, which allows medium perfusion of the leak-tight tubes (Schutgens et al. 2019; Gijzen et al. 2021). This system could be adapted to investigate the effect of crystal-mediated injury on tubuloid cell growth, cytokine production, and barrier integrity. Tubuloids could be used to further determine the effects of crystals on epithelial cells and reciprocal effects for crystal retention as well as progression to nephrolithiasis. Co-cultures of crystal injured tubuloid cells with macrophages or dendritic cells could provide further insight in how tubular injury affects local immune responses and renal inflammation (Ng et al. 2008; Flach et al. 2011; Franklin et al. 2016).

Acute interstitial nephritis is a primarily drug-induced—e.g., by antibiotics or non-steroidal anti-inflammatory drugs—T cell-mediated type-4 delayed hypersensitivity. Even after removal of the drug, many patients do not recover their baseline kidney function. Steroid treatment within 7 days after diagnosis is proposed to improve the outcome further indicating the role of immune cells in the pathogenesis (Praga and González 2010). iPSC-derived kidney organoid and tubuloid models established from patients and co-cultured with autologous T cells could be used to assess the effect of steroids and other anti-inflammatory drugs on tubular regeneration. At present, an organoid model of acute interstitial nephritis does not exist yet, however, the fast invasion and expansion of immune cells could be modeled in a co-culture system. Nephrotoxicity of drugs including gentamicin and cisplatin has already been successfully modelled in kidney organoids, which supports their utility for drug-testing in a patient-specific in vitro model (Morizane et al. 2015). In summary, iPSC-derived kidney organoids and tubuloids hold potential to study the role of immune cells in several kidney diseases. Recent advances in gene-editing of iPSC-derived kidney organoids and tubuloids with CRISPR/Cas 9 would allow to pinpoint specific immune pathways mediating pathogenesis in kidney diseases (Freedman et al. 2015; Forbes et al. 2018; Przepiorski et al. 2018; Calandrini et al. 2020; Schene et al. 2020). However, further studies are required to better characterize the cells in iPSC-derived kidney organoids and tubuloids and most importantly increase cellular complexity and maturation to develop meaningful in vitro models of immune-mediated kidney diseases.

## Kidney organoids and tubuloids to model immune-mediated renal tissue regeneration

In the last years, the immune system’s role in tissue repair mechanisms is increasingly appreciated (Cressman et al. 1996; DeAngelis et al. 2012; Karin and Clevers 2016). The influence of immune cell-derived cytokines on tissue development, regeneration, and inflammation has been investigated in several organoid systems (Bar-Ephraim et al. 2020). Recently, we employed intestinal organoids to study the development of necrotizing enterocolitis in preterm infants and developed, after careful medium assessment and adaptation, a co-culture system of autologous intestinal CD4^+^ T cells and intestinal organoids. These studies revealed that TNFα produced by fetal intestinal CD69^+^ CD4^+^ T effector memory cells promotes epithelial stem cell proliferation and aids the development of the fetal intestine, but can also mediate intestinal inflammation upon premature birth (Schreurs et al. 2019). This study illustrates that organoid-immune cell co-cultures can be used to model inflammatory diseases and immune-mediated instruction of fetal tissue development. Similarly, iPSC-derived kidney organoids recapitulating nephrogenesis could be used to study the influence of immune cell-derived cytokines on kidney tissue development. Furthermore, as tubular cells are primarily affected in acute kidney injury, co-cultures of tubuloids with CD4^+^ T cells would allow to investigate their role in facilitating tissue regeneration and repair. Notably, IL-4—a pleiotropic cytokine initially recognized for its role in inflammation and type 2 immunity—also promotes epithelial cell proliferation in several tissues (Stein et al. 1992; Voehringer et al. 2006; Biton et al. 2018; Cortes-Selva et al. 2019; Jayme et al. 2020). IL-4 promotes GATA3 signaling in T cells (Shao et al. 2008; Feng et al. 2018), and GATA3 signaling is also critical for renal development suggesting a potential role to aid tissue regeneration (Grote et al. 2006). At the same time, IL-4 is known to contribute to renal fibrosis and can thereby decrease kidney tissue functionality (Shao et al. 2008; Feng et al. 2018). Co-cultures of intestinal organoids with IL-22-producing innate lymphocytes have further illustrated the instruction of tissue repair by immune cells via IL-22-induced STAT3 signaling (Lindemans et al. 2015). These observations provide support to use in vitro kidney organoid models for studies of the immune system’s role in tissue regeneration and proliferation. Taken together, iPSC-derived kidney organoids recapitulating nephrogenesis as well as tubuloids recapitulating renal tissue repair can be used to study the influence of cytokines on renal tissue development and regeneration.

The complications of tissue injury and reduced tissue regeneration are important contributors to morbidity in patients suffering from kidney diseases. The long-term consequences of tissue damage are particularly apparent in the kidney’s filtration system due to the low regenerative capacity, especially of podocytes (Nagata et al. 1998; Wanner et al. 2014; Shankland et al. 2014). The tubular compartment, albeit being capable of dedifferentiation and repopulation of damaged structures, can also be overcome by severe or prolonged disease courses, leading to interstitial fibrosis and tubular atrophy. This is reflected by the need for a kidney transplant or dialysis in case of terminal renal insufficiency. Enhancing the proliferative capacity and tissue repair of kidney epithelial cells would be a critical advancement in development of novel therapeutic approaches to prevent renal failure. For example, macrophages are proposed to contribute to reparative mechanisms in several tissues including the kidney and exhibit remarkable plasticity to acquire these repair features (Stein et al. 1992; Stout and Suttles 2004; Nelson et al. 2012). Depending on micro-environmental cues, renal macrophages can differentiate into functionally different subsets expressing a range of pro-inflammatory, anti-inflammatory or reparative factors (Kluth et al. 2001; Lin et al. 2009; Vernon et al. 2010; Cao et al. 2010; Nelson et al. 2012). Macrophages have already been co-cultured with control and irradiated organoids of mammary glands. Live-cell fluorescence imaging revealed that macrophages preferentially migrate into irradiated organoids (Hacker et al. 2019). Co-culturing iPSC-derived kidney organoids and tubuloids with macrophages would be an interesting approach to not only assess the influence of macrophages on tissue growth but also investigate kidney epithelium-mediated macrophage activation and differentiation. On the other hand, macrophages can contribute to the progression of several renal diseases (Qi et al. 2008; Wang et al. 2008; Hu et al. 2019; Zimmerman et al. 2019; Torres et al. 2020). Recently, the role of IL-1ß produced by macrophages and DCs has been studied in tubulointerstitial fibrosis using iPSC-derived kidney organoids. Organoids incubated with IL1-ß revealed cytokine-dependent hypertrophy, proximal tubule injury, and fibrosis with collagen I deposition (Lemos et al. 2018). These studies illustrate, using an iPSC-derived kidney organoid model, the strength of organoids to study the effects of dysregulated immune responses in kidney disease.

In summary, co-cultures of organoids and immune cells or immune cell-derived cytokines have been used to successfully model and assess the interactions between epithelial cells and components of the immune system, with pioneering studies using iPSC-derived kidney organoids indicating the possibilities offered by organoids to study immune-mediated regulation of renal tissue development, regeneration, and inflammation.

## Current limitations of organoid-immune cell co-cultures

**Fig. 3:**
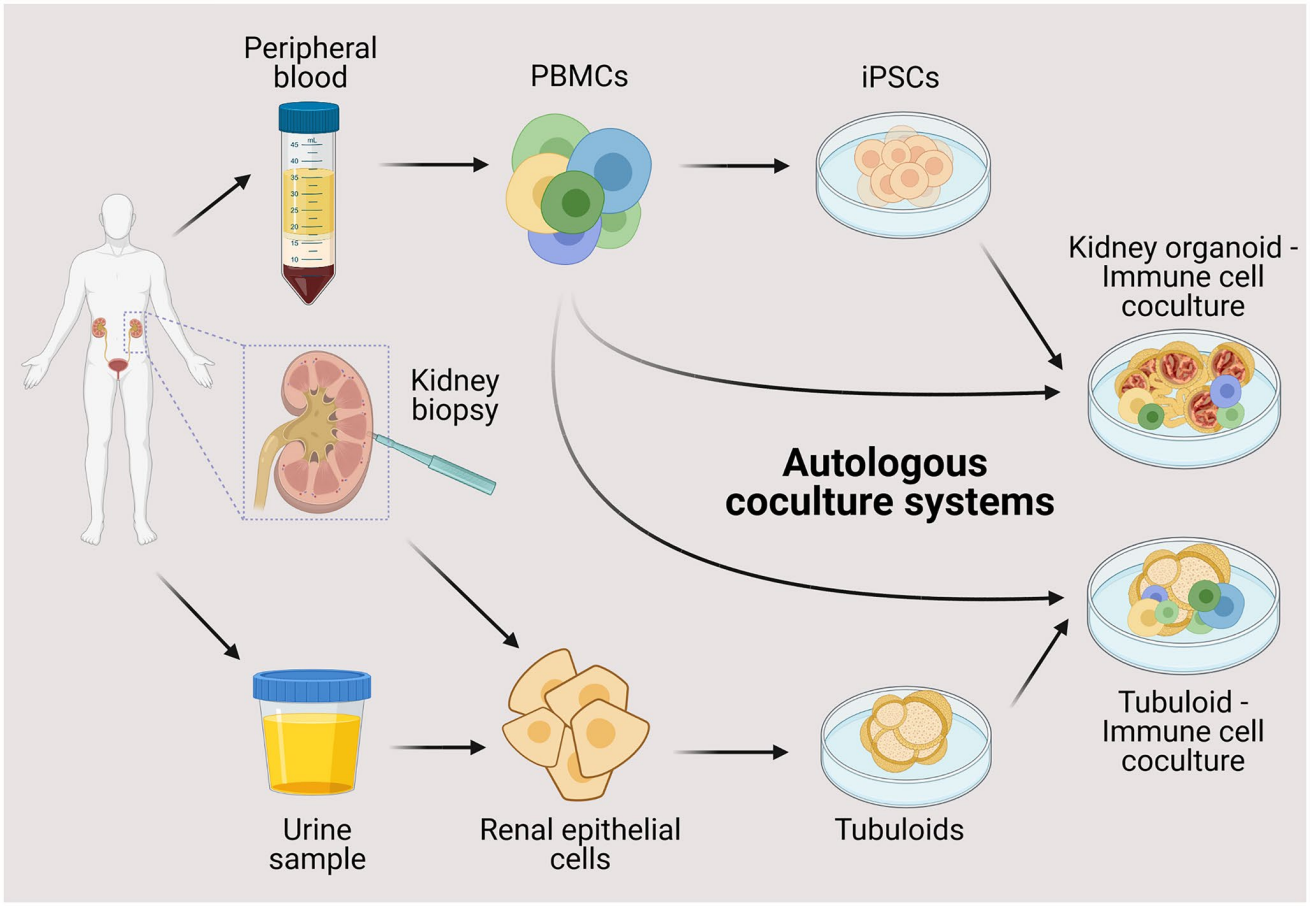
Future approaches for autologous organoid–immune cell co-culture systems

At present, several challenges that impede the co-culture of iPSC-derived kidney organoids and tubuloids with immune cells exist. Foremost, the propagation of iPSC-derived kidney organoids and tubuloids requires a complex medium enriched with growth factors and nutrients (Morizane et al. 2015; Takasato et al. 2015; Schutgens et al. 2019). Immune cells can exhibit receptors for these medium components, which influence their activation and differentiation. For example, A8301 is an inhibitor of TGF-β (transforming-growth-factor beta) signaling via ALK4, ALK5, and ALK7 as well as Smad2. TGF-ß signaling impacts T helper cell polarization; therefore, responses may be altered when T cells are co-cultured in tubuloid medium (Tojo et al. 2005; Xu et al. 2009; Krebs and Panzer 2018). Differentiation of tubuloids however occurs in growth factor reduced medium (Schutgens et al. 2019), providing a potential strategy to avoid medium-dependent activation and polarization of immune cells. Other factors added to organoid medium such as fibroblast growth factors may also impact immune cell functioning (Workalemahu et al. 2004). After the induction of iPSC-derived kidney organoids, they are cultured in growth factor free medium; clearly, this will simplify co-cultures with immune cells and will enable studies investigating interactions with immune cells in these more advanced stages of kidney organoids. On the other hand, immune cell cultivation also requires certain media components, which in return might impact organoid cells. IL-2 is an important component of T cell culture medium (Cantrell and Smith 1984; Stern and Smith 1986). A recent study revealed that podocytes also express a functional IL-2 receptor, whose activation results in podocyte injury, apoptosis, and mitochondrial depolarization (Zea et al. 2016). In sum, the application of kidney organoids and tubuloids in co-cultures with immune cells requires careful assessment of media components to rule out possible bias and design meaningful studies. Furthermore, the organoid’s and immune cell’s origin in terms of their HLA background needs to be taken into consideration. Allogenic immune responses have been shown to underlie severe inflammation in kidney transplants and the combination of organoids and immune cells from different donors can impact results observed in co-cultures. At the same time, kidney organoid systems hold the potential to be used in autologous organoid-immune cell systems in which all cells are derived from the same donor (Fig. 3). Patient-derived PBMCs can serve as a source for iPSCs (Zhou et al. 2012; Sommer et al. 2012) as well as for immune cells, providing a source to generate autologous co-cultures. Similarly, the co-culture of urine- or tissue-derived tubuloids with blood-derived immune cells from the same patient offers another possibility to model kidney diseases in an autologous system paving the way for personalized medicine.

## Conclusion

Complex interactions between epithelial cells and cells of the immune system play a key role in immune-mediated pathologies. Recently developed iPSC-derived kidney organoids and tubuloids both hold potential for future research of immune-mediated kidney diseases. iPSC-derived kidney organoids are well-suited models to accurately represent several aspects of the developing human kidney, providing further insight into nephrogenesis and congenital anomalies. Notably, novel therapeutic strategies are currently tested in organoid models (Dijkstra et al. 2018; Hollywood et al. 2020). Further development of iPSC-derived kidney organoids is required to advance these models to represent mature kidney tissue. These matured organoid models would potentially be better suited to uncover the role of immune cells in the regulation of renal cell differentiation and kidney diseases. Tubuloids are a less complex model of the kidney as they lack the glomerulus with its cellular components. Nevertheless, due to their rapid establishment and genetic stability, representing the donor’s genotype and phenotype over long culture periods, tubuloids hold potential for patient-specific modeling of diseases and personalized medicine. With recent advances in the co-culture of organoids and immune cells, new perspectives for modeling immune-mediated kidney diseases arise. The implementation of autologous organoid systems containing renal cells and immune cells from the same donor will provide a unique model opening up new avenues to uncover fundamental principles governing the interactions of immune cells and renal epithelial cells in health and disease. Nevertheless, the application of iPSC-derived kidney organoids and tubuloids to study the mechanisms underlying several kidney diseases requires further adaptions of current models. Building upon recent advances made within other organoid systems, the developments needed to design robust kidney organoid-immune cell co-culture systems can be propelled forward. In conclusion, iPSC-derived kidney organoids and tubuloids provide a novel platform for studies of immune-mediated instruction of kidney development, tissue regeneration, and inflammation.
